# Supplementary material for: Neural oscillations in the fronto-striatal network predict vocal output in bats
Source: PLoS Biol. 2020 Mar 19;18(3):e3000658. doi: 10.1371/journal.pbio.3000658 (PMC7081985; doi:10.1371/journal.pbio.3000658)
Supplement: Attachment — Submitted filename: response letter.docx [file pbio.3000658.s013.docx]

Copy of decision letter.

Dear Dr Hechavarria,

Thank you very much for submitting your manuscript "Neural oscillations in the fronto-striatal network predict vocal output in bats" for consideration as a Research Article at PLOS Biology. Your manuscript has been evaluated by the PLOS Biology editors, an Academic Editor with relevant expertise, and by three independent reviewers.

You’ll see that all three reviewers are largely positive about your study, but raise concerns about the framing (rev #1) and the nature of the distinction between different call types and their corresponding oscillations (revs #2 and #3). There are a substantial number of further requests for presentational improvement and additional analyses; all of the issues raised by the reviewers should be addressed for further consideration.

In light of the reviews (below), we will not be able to accept the current version of the manuscript, but we would welcome resubmission of a much-revised version that takes into account the reviewers' comments. We cannot make any decision about publication until we have seen the revised manuscript and your response to the reviewers' comments. Your revised manuscript is also likely to be sent for further evaluation by the reviewers.

Your revisions should address the specific points made by each reviewer. Please submit a file detailing your responses to the editorial requests and a point-by-point response to all of the reviewers' comments that indicates the changes you have made to the manuscript. In addition to a clean copy of the manuscript, please upload a 'track-changes' version of your manuscript that specifies the edits made. This should be uploaded as a "Related" file type. You should also cite any additional relevant literature that has been published since the original submission and mention any additional citations in your response.

Please note while forming your response, if your article is accepted, you may have the opportunity to make the peer review history publicly available. The record will include editor decision letters (with reviews) and your responses to reviewer comments. If eligible, we will contact you to opt in or out.

Before you revise your manuscript, please review the following PLOS policy and formatting requirements checklist PDF: <http://journals.plos.org/plosbiology/s/file?id=9411/plos-biology-formatting-checklist.pdf>. It is helpful if you format your revision according to our requirements - should your paper subsequently be accepted, this will save time at the acceptance stage.

Please note that as a condition of publication PLOS' data policy (<http://journals.plos.org/plosbiology/s/data-availability>) requires that you make available all data used to draw the conclusions arrived at in your manuscript. If you have not already done so, you must include any data used in your manuscript either in appropriate repositories, within the body of the manuscript, or as supporting information (N.B. this includes any numerical values that were used to generate graphs, histograms etc.). For an example see here: <http://www.plosbiology.org/article/info%3Adoi%2F10.1371%2Fjournal.pbio.1001908#s5>.

For manuscripts submitted on or after 1st July 2019, we require the original, uncropped and minimally adjusted images supporting all blot and gel results reported in an article's figures or Supporting Information files. We will require these files before a manuscript can be accepted so please prepare them now, if you have not already uploaded them. Please carefully read our guidelines for how to prepare and upload this data: <https://journals.plos.org/plosbiology/s/figures#loc-blot-and-gel-reporting-requirements>.

Upon resubmission, the editors will assess your revision and if the editors and Academic Editor feel that the revised manuscript remains appropriate for the journal, we will send the manuscript for re-review. We aim to consult the same Academic Editor and reviewers for revised manuscripts but may consult others if needed.

We expect to receive your revised manuscript within two months. Please email us ([plosbiology@plos.org](mailto:plosbiology@plos.org)) to discuss this if you have any questions or concerns, or would like to request an extension. At this stage, your manuscript remains formally under active consideration at our journal; please notify us by email if you do not wish to submit a revision and instead wish to pursue publication elsewhere, so that we may end consideration of the manuscript at PLOS Biology.

When you are ready to submit a revised version of your manuscript, please go to <https://www.editorialmanager.com/pbiology/> and log in as an Author. Click the link labelled 'Submissions Needing Revision' where you will find your submission record.

Thank you again for your submission to our journal. We hope that our editorial process has been constructive thus far, and we welcome your feedback at any time. Please don't hesitate to contact us if you have any questions or comments.

Sincerely,

Roli Roberts

Roland G Roberts, PhD,

Senior Editor

PLOS Biology

*****************************************************

REVIEWERS' COMMENTS:

**Reviewer #1:**

This manuscript describes a series of experiments in which local field potentials were simultaneously recorded in the striatum and frontal cortex of bats, and the patterns of neural activity were analyzed and compared before and after vocalizations were emitted. The manuscript focuses on comparing neural activity across two different types of vocalizations, echolocation calls versus communication calls. The LFPs are characterized and segregated by frequency into low (theta), medium (beta) and high (gamma) band activity. The study provides solid evidence of a functional interaction between neuronal activity patterns in frontal cortex and striatum, and found that activity patterns were different between the two types of vocalizations. Overall, the study is original, creative and the results are interesting. The presence of coherent premotor neural activity patterns in the frontal cortex and striatum preceding vocalizing is especially important since the role of the basal ganglia in non-human mammalian vocalizations remains poorly understood. The differences in the post-vocalization activity, which appears auditory in origin, are also quite intriguing.

My main criticism of the manuscript is that the hypothesis is not well justified, and the rationale for the study and its interpretation is vague and confusing. Most of the references cited in the introduction to build the rationale come from human speech and primate communication studies (3-14), with very little support for the presence of analogous brain networks in bats or other mammals (or songbirds). The stated hypothesis (Intro, 4th paragraph) is that the production of echolocation and non-echolocation (communication) calls could involve different fronto-striatal oscillatory dynamics. Predicting that the rhythms might be different during different behaviors isn’t a very satisfying test of the hypothesis unless the experiments is designed to reveal something specific about the underlying mechanism. The potential significance of different oscillatory frequencies or states for cognition and motor control is only generally addressed, and consequently the interpretation lacks any sense of mechanism. What seems to be absent is a cogent connection between corticostriatal oscillatory network activities and the mammalian (vocal) motor pathways. Without this, it is difficult to evaluate the significance of these results.

We thank the reviewer for taking the time to evaluate our manuscript and for these insightful comments. We agree in that most of the literature cited in the previous MS version and the hypothesis regarding the role of fronto-striatal oscillations on vocal control was backed up mostly using primate data. The reason for the latter is that most studies relating basal ganglia and frontal cortex to vocal production were conducted either in humans or non-human primates.

That said, the reviewer is absolutely right in that there is no hard evidence indicating that fronto-striatal circuits and their dynamics are preserved in the vertebrate clade. We conducted a thorough literature search and cited several other studies addressing the link between fronto-striatal regions to the canonical vocal motor pathways and dealing with the putative role of corticostriatal area in vocalization in humans, bats and songbirds (p. 3). We were unable to find studies relating fronto-striatal oscillations to vocal output in rodents. If the referee is aware of such studies, we would of course gladly add them to the cited literature.

In the introduction, we wrote:

*In this article, we studied neural activity in the dorsal striatum (caudate nucleus, CN) and frontal cortex during vocalization in bats. We chose to study the fronto-striatal circuit because there is strong evidence suggesting a role of this network in vocal production across vertebrate species. Fronto-striatal networks connect different parts of the frontal lobe with various regions of the striatum which constitute a major input structure into the basal ganglia [7, 8]. Using tractographic methods, a direct connection between the CN with the prefrontal cortex (PFC) has been identified in humans and other mammals [9-12]. Both brain regions are highly connected to brain areas of the canonical vocal motor pathway. For example, in primates, the caudate nucleus receives inputs from the laryngeal motor cortex [13, 14]. It is also known that the frontal cortex is connected to structures participating in vocal control such as the periaqueductal grey in the brainstem [15]. Moreover, studies examining the function of frontal and striatal regions, have identified their putative role in vocalization in humans [16-18] and bats [19, 20]. Likewise, in songbirds, Area X (the bird striatum) appears to be involved in vocal learning and in modulating song production in adult animals [21]. Together these studies support the involvement of frontal and striatal areas in mediating and predicting vocal output across vertebrate species.*

Specific comments

Intro page 2, line 5: what is the “hearing-action cycle”?

The hearing-action cycle refers to the interconnection between hearing and action, i.e. running after hearing the word danger or uttering words to reply to a question. It is true that this is not a widely used term. For better comprehension, we changed the “hearing-action cycle” to “hearing and vocal production”.

Line 7: distress and social calls are communication calls. Why use the term non-echolocation rather than communication throughout the paper?

Thank you for this important comment. We used the term non-echolocation calls to clearly distinguish between echolocation pulses and the “remaining” broad type of vocalizations. We agree that it might be confusing and thus changed it to “communication calls” throughout the manuscript.

Results, 2nd paragraph: For data analysis it makes sense to focus on utterances surrounded by a +/- 500 ms time window of silence, but wouldn’t this exclude normal echolocation behavior in which calls emitted in rapid trains? Don’t bats normally emit echolocation calls more frequently than once per second?

This is true, especially when bats are approaching an object/obstacle. However, as shown in our manuscript, head-fixed bats also produce isolated echolocation pulses. As producing more than one vocalization within a short time interval may lead to (different) footprints in the neural data, we decided to analyze only isolated vocalizations.

In this manuscript version we specify that future studies should look into the neural activity patterns leading to the production of echolocation trains. We wrote (Discussion p. 17 second paragraph).

*Note that in the present study, we focused on analyzing oscillatory activity related to vocalizations that were surrounded by silent periods to avoid possible confounds related to the production of calls trains (except in Fig. 6c, were calls with contaminated post-vocalization times were used as control for the prediction analysis). We reasoned that situations in which bats produced vocalization trains with mixtures of echolocation and communication calls could render misleading results. Future studies could assess whether prevocalization activity carries information about the physical parameters of vocalization trains.*

Why did the bats emit communication calls if they were all alone in the recording chamber? Was there a method for evoking calls?

Thanks for this question. There was no special method for evoking communication calls. Occasionally (~ every 10 min) the door of the Faraday chamber was opened to stimulate vocalization production.

Our data does not allow to assess the reason why bats vocalize in isolated environments but one could speculate. One possible explanation could be that bats are trying to assess if conspecifics respond to their vocal emissions (i.e. searching for an audience?) or are trying to inform other individuals about their location etc... It is impossible for us to know which of the latter (if any) explains vocal production in our recording settings. We feel that including our own speculations in the manuscript could bias readers, but we are of course prepared to include them at the referee’s request.

Results, 4th paragraph: The acoustic responses are puzzling. Line 5 says LFPs in both regions revealed a preference towards low frequencies (i.e those characteristic of the communication calls). If these neuronal populations weren’t very responsive to the echolocation call frequencies, why would they be involved in echolocation call generation?

Thanks for this important comment. A preference towards low frequencies refers only to the “best frequency” measured with pure tones. The latter does not imply that these brain areas are not responsive to echolocation sounds which have a much more complex spectro-temporal design than a simple pure tone. In fact, we did observe strong responses following vocalization production in both the communication and echolocation situations (Fig. 2).

This is now specified in page 8 (end of second paragraph of this MS version).

*Note that a preference towards low frequency sounds does not necessarily imply a lack of responses to natural high frequency sounds such as echolocation calls (see below).*

Page 8, first line: the observed effects would seem to support only a correlation between LFP frequencies and different vocalizations. A functional or causal role doesn’t seem to be supported yet.

Thank you for this important comment. Indeed, we do not know if there is as causal relationship. We changed the sentence to “*Both effects observed suggest that the power in distinct striatal LFP frequencies can be correlated with the production of different types of vocalization (beta higher for echolocation and gamma for communication).”* (p.9 last paragraph). We also removed al statements indicating causality throughout the manuscript

Discussion, 2nd paragraph. Much of the discussion, but especially this paragraph (lines 9-19) seem highly speculative to me.

Thank you for this comment. We tried to improve the discussion (and especially this paragraph) and changed/deleted some parts. In particular, we changed this paragraph (in this ms version p. 19 last paragraph) to:

“*Differences in LFP activity preceding vocal production also occurred in the beta band. According to our data, in bats, the beta band of LFPs is differentially involved in echolocation pulse and communication call emission. Beta power is highest during echolocation production in the CN and in superficial layers of the FAF (see Figs. 3 and 4). As especially the beta band activity is correlated with motor action planning and performance [36, 37], one could hypothesize that the strength of beta oscillations in the CN and in superficial FAF layers is linked to different sensory-motor programs required for the production and/or post-vocalization evaluation of acoustic signals (i.e. echoes during echolocation). Overall, beta is typically dominant in the motor system, correlating with the maintenance of ongoing sensorimotor cognitive states and endogenous timing processes [35, 76]. Aberrant beta oscillations (especially in the striatum) are also a key feature of the parkinsonian brain [4, 8, 77]. Our results, together with those from previous studies suggest that beta oscillations within the fronto-striatal path are important for vocal motor output production.*

*Another large vocalization-type dependent effect was detected in the gamma band. Before echolocation, high power in this frequency band was observed in deep layers of the FAF, whereas before communication high gamma power was found in the CN. As the power maxima in gamma was reversed across vocalization conditions in both brain structures, it could be suggested that each component of the fronto-striatal path relies on a differential power distribution of high frequencies in order to produce the same vocal output. This could be supported by the fact that in both brain structures power in the gamma band was the best predictor of vocal output (Fig. 6).*”

Page 14, line 19: “One possible explanation could be that echolocation calls require a more thorough sensory processing after vocal production than non-echolocation calls”. This would seem to go without saying, but it doesn’t really explain why there wasn’t an auditory response to the self-heard communication calls. Were there auditory responses to the acoustic stimuli, but no auditory responses during the communication call utterances?

It is true, that there should still be an auditory response following communication calls. This sentence did not refer to the auditory response after vocalization production per se, but to the coherence between FAF and CN. The lack of late fronto-striatal coherence after communication calls does not imply that there is no response in either of the structures, only that responses are not temporally linked at time periods after 250 ms post-vocalization, as it was the case for echolocation. In fact, as it can be seen in Fig. 2 there was a strong LFP response following communication call production.

To make the sentence better understandable we changed it to “*One possible explanation for the strong coherence following echolocation pulses could be that the latter require a more thorough sensory processing and auditory feedback after vocal production (i.e. for echo evaluation) than communication calls.*”. (p. 19, 1^st^ paragraph)

Page 16, line 7: The results don’t fully support the conclusion that neural activity patterns in FAF or CN can “represent future vocal outputs”, only that there is a correlation between network activity patterns and broad categories of vocal output, which may or may not be directly affiliated with motor control.

Thank you, that is correct. We changed the sentence to: “*Our results show that not only the FAF, but also the gamma power in the CN is correlated with the type of vocal output*.” (p. 21, 2^nd^ paragraph)

**Reviewer #2:**

General summary: Understanding the neural circuits underlying the production of vocal signals is an important endeavor, especially because humans are so reliant on speech and language for communication. In this study, Weineck et al. record neural activity in the frontal cortex and striatum in bats producing echolocation and non-echolocation calls. They find that neural activity and patterns of coherence in and between these brain areas varies between echolocation and non-echolocation calls. These are novel findings that have implications for the control of communication.

Major comments:

It is important for readers to fully understand the extent of acoustic differences between echolocation (E) and non-echolocation (NE) calls included in their analyses. In this respect, it will be very important for the authors to depict, on the same figure, E and NE calls using both duration and peak frequency (2-D image). Based on the histograms, there is considerable overlap in both duration and peak frequencies, but these vocalizations could appear more distinct if each call is depicted using both features. One could also include frequency modulation as a third feature to help distinguish between E and NE calls.

Overall, the NE class of vocalizations is quite broad. The authors note in the Introduction that NE calls include communication, distress and social calls. It is unclear how acoustically distinct these types of NE calls are, or to what extent the NE calls in the analyses reflect these different types of NE calls. Given the differences between E and NE calls, it is possible that different types of NE calls are driven by different patterns of activity and coherence in the brain. Therefore, it seems important for the authors to parse their dataset of NE calls into different types of communication signals. One could also image it being interesting to assess the degree to which variation in signal properties within NE (and E) calls are related to LFP activity. At minimum, the authors should discuss the range of NE calls produced, the possibility of multiple types of NE calls emitted by the bats, and the limitations of their interpretations given the range of NE calls that could have been emitted during neurophysiological recordings.

Thank you for these valuable comments.

A graphic representation combining peak frequency and duration in communication and echolocation calls is shown in Fig. 1f of this manuscript version. Note that we recalculated peak frequency and duration of each call to double check that the automatic measurement algorithm from Avisoft was not providing any errors. In the current figure, one can clearly assess the dissociation between echolocation and communication sounds in the frequency domain. This is also obvious in median spectra of the two call types represented in Fig. 1g.

Regarding communication call variability, you are totally right. Communication calls (=NE calls in the previous version) exhibit large variability and can occur in different behavioral contexts such as e.g. mating, distress calling, fights between individuals, etc. It is difficult to disentangle the different types of communication calls based on our dataset given the simplicity of our experimental paradigm in terms of behavior. In other words, one would need to score the bats’ behavior to get an idea of the meaning/type of the communication call produced. We fear that parsing the calls recorded based solely on physical attributes could lead to data misinterpretation.

That said, in this version of the manuscript we do bring the readers’ attention to the fact that the communication call category is quite broad (Discussion, p. 17 2^nd^ aragraph). We wrote:

*Overall, the observed electrophysiological effects during communication call production need to be considered cautiously. Bats produce communication calls in numerous situations such as e.g distress calling, courtship, territorial disputes, among others [44, 45]. The most common way to parse communication calls into different subcategories is to score the behavior/context during which the calls are produced [59, 71, 72]. As we recorded in head-fixed animals, it is difficult to assess what type of communication calls were broadcasted. Pooling vocalization trials from many types of communication calls together could potentially hide call specific effects.*

In addition, based on comments made by reviewer 3, we were able to parse our communication dataset into two broad groups: one group of communication calls with only high power at low frequencies (<50Hz), whereas the second group additionally showed pronounced power in high frequencies (>50 kHz). We ran the same analysis for these two subcategories of communication calls to assess the underlying mechanism of call production. The results can be found in Fig. 5 and Supplementary Fig. 6. As it can be seen, we were able to replicate our results even after subdividing the communication group. Regarding this new analysis, in the discussion we wrote (end of page 17):

*Based on spectro-temporal features we identified two types of communication vocalizations (containing either only low frequency or high and low frequency components, see Supplementary Figures 2 and 8). Even communication calls containing pronounced power at high frequency differed markedly from echolocation vocalizations. The latter indicates that differences observed in terms of LFP spectral structure and inter-areal coupling are not related solely to the absence/presence of high frequencies in the sounds uttered. Note, that we cannot discard that communication calls containing only high frequencies involve LFP patterns similar to those observed during echolocation. Communication calls carrying only high frequencies were not observed in our dataset, but C. perspicillata produces such calls in contexts such as mating [59].*

Related to this point, the authors should comment on the range of peak frequencies observed in E calls. The range seems quite large and it is important for a broad audience to understand the extent of this variation.

Thanks for pointing this out. As mentioned in response to one of the previous comments, we double checked the values of peak frequency and call duration for each of the syllables to be sure there were no errors. We noticed that the Avisoft software was miss-calculating peak frequency in some of the calls, in situations in which the ratio acoustic-noise vs. emitted sound favored the former. This mistake was causing some echolocation calls to be tagged as low peak frequency, which was not correct. To obtain a more accurate peak frequency estimate for each call, we de-noised the call’s FFT by subtracting the FFT of the noise floor (call duration and noise estimates were matched in each case). Then, the frequency of highest power in the denoised FFT was taken as peak frequency. A description of the FFT denoising algorithm was added to the methods of this MS version (p. 25 last paragraph).

We did notice that even after de-noising there was some variability in the E-call category. Regarding this point, we wrote the following in the results section (page 7, 2^nd^ paragraph):

*In the echolocation category, differences in peak frequency across calls could be due to inter-individual variability in sonar pulse design and to the use of different echolocation harmonics even by the same bat.*

One of the most consistent results in their paper is that many changes in neural activity and coherence are found after vocalizations. The authors explicitly acknowledge the prevalence of these results and suggest that these neural changes reflect processing of auditory feedback. While understanding the nature of auditory responses outside the context of vocalization is distinct from understanding the nature of auditory feedback processing, it would be very informative for the authors to conduct the same level of analyses of their data on auditory responses (e.g., LFP deflections, oscillations, coherence). This is useful even if the authors did not present a stimulus set that reflected the range of vocalizations emitted by bats. (If there are limitations on space, the authors could move much of the machine learning results to the Supplementary Information.)

Thanks for this insightful comment. We agree with the referee that post-vocalization activity is very interesting. We would like to point out that even though the previous version of the manuscript had a strong focus on pre-vocalization signals, most of our analyses also included post-vocalization time. The LFP plots (Fig. 2), the spectral differences analysis (Fig. 3-5, this manuscript version), and the coherence analysis (Fig. 7) display time on the x-axis ranging from -500 to +500 after vocalization.

In addition, following the referee’s suggestions, for this manuscript version we ran additional analysis for the post-vocalization time using the support vector machine classifier (Fig. 6b of this MS version). In a nutshell, we observed that classifying vocalizations based on post-vocal activity was not much more accurate than the results obtained using pre-vocal signals. The latter emphasizes the strong correlate between prevocal neural activity and vocal outputs. This information is now described in the results (p. 12, 2^nd^ paragraph). We wrote:

*Next, the same SVM classifier analysis was performed based on the power of LFPs recorded after vocal production (Fig. 6b). For the post-vocalization LFP power, the best prediction occurred again in deep layers of the FAF in gamma frequencies (maximum of ~78% correct hits). Interestingly, prediction power in the CN was lower in post-vocalization signals when compared to the pre-vocalization time (compare colors in top panels of Fig. 6a-b). The latter suggests that evoked responses following vocal production in the striatum are poorly correlated with the type of vocalization perceived by the bats.*

The Introduction should provide more information on the potential importance of rhythms and coherence for action selection. I think much of the details about coherence and rhythms that is currently in the Discussion section should be moved to the Introduction. This will allow the reader to process the many types of data in the Results section.

Thank you for this useful comment. We moved parts of the discussion to the introduction and explained brain rhythms and the potential function of different frequency bands in a separate paragraph (p. 4).

*In this study, synchronized neural oscillations occurring in the LFPs before and after vocal production To assess the neural dynamics during vocalization, we recorded local field potentials (LFPs) and spiking activity during the production of echolocation pulses and communication calls in bats. LFPs reflect the sum of synaptic activity in neuronal populations and slow spike components and they represent a correlate of signals obtained with non-invasive techniques such as electroencephalography [22]. We focussed on investigating synchronized neural oscillations occurring in the LFPs before and after vocal production. Oscillations are thought to enable communication between neural populations and, at least in humans and birds, they are known to be related to vocal production [23-25].*

*Neural oscillations can be split into different frequency bands comprising delta (1-3Hz), theta (4-8Hz), alpha (8-12Hz), beta (12-30 Hz) and gamma (30-80Hz). Empirical evidence indicates that LFP oscillations with different frequencies correlate well with distinct neural computations, motor control and cognitive states [26]. In particular, low frequencies such as theta and alpha are known to modulate sensory processing, action selection and neuronal excitability; are implicated in cognitive control and are involved in long range synchrony facilitating e. g. top-down processing [27-32]. Beta band oscillations potentially hold functions in perception, memory and sensory processing [33-35], are linked to motor actions in the motor cortex and striatum [36, 37] and are dysregulated in disorders such as Parkinson’s disease [6, 38]. Gamma rhythms can be linked to selective attention, (local) neural computation, motor control [25, 27, 39] and are correlated with vocalization production [24, 40]. In humans, distinct oscillatory patterns and coherence across frequency bands have been found during speech production and singing [41, 42].*

The authors have plenty of additional data to analyze, since their (sensible) criteria for call selection dramatically winnowed down the number of calls for analysis. However, given their emphasis on elucidate neural activity that could lead to call production, it would be useful to independently analyze the data for echolocation and non-echolocation calls that were preceded by 500 ms of silence but did not have to be followed by 500 ms of silence. I think this would be a very useful dataset to test the robustness of their findings.

Again, thank you very much for this suggestion. We used the remaining vocalization dataset (the one with contaminated post-vocal activity) to test the SVM classifier results (Fig. 6c). This is a good control inspired on this reviewer comments and it emphasizes the robustness of the results presented in this manuscript. We wrote (page 12, 3^rd^ paragraph):

*In a last step, we ran the classifier analysis using a third dataset composed of pre-vocalization LFPs in trials in which the post-vocalization time was contaminated with other sounds produced by the animal. Note this includes cases in which trains of vocalizations (sometimes mixtures of echolocation and communication) were produced. Despite this possible confound, the classifier was still able to reach accuracy levels of ~68% correct hits when considering gamma activity in deep FAF channels (Fig. 6c).*

We also added the following to the discussion (page 17, 2^nd^ paragraph):

*Note that in the present study, we focused on analyzing oscillatory activity related to vocalizations that were surrounded by silent periods to avoid possible confounds related to the production of calls trains (except in Fig. 6c, were calls with contaminated post-vocalization times were used as control for the prediction analysis). We reasoned that situations in which bats produced vocalization trains with mixtures of echolocation and communication calls could render misleading results. Future studies could assess whether prevocalization activity carries information about the physical parameters of vocalization trains.*

The timing of the increase in LFP signals for echolocation calls is very late for auditory processing of a fast signal for detection objects in the world (Figure 2d). And the peak in coherence between the FAF and CN is even later, making it even less likely to be involved in auditory processing. The latency is also longer than that observed for auditory response to pure tones (Supplementary Figure 2). Please respond to this.

This is absolutely true. The latencies are too long for immediate auditory processing, especially for echolocation pulses. However, we should emphasize that the fronto-striatal path does not belong to the canonical sound-analysis network, resulting in potentially longer latencies than other brain structures such as the auditory cortex.

In terms of latency, probably one could distinguish between early auditory-evoked (short latency) responses and late activity which could putatively represent auditory feedback. One hypothesis could be that vocal production involves a sensitive “window” right after call emission for receiving auditory feedback which could be useful for evaluating the result of the sound emitted; i.e. the echo delay in the case of echolocation or the response of other individuals (or their absence thereof) to the sound emitted. We added the following to the manuscript (page 19, 1^st^ paragraph):

*… One possible explanation for the strong coherence following echolocation pulses could be that the latter require a more thorough sensory processing and auditory feedback after vocal production (i.e. for echo evaluation) than communication calls. Such post-processing of echolocation pulses could be relevant for planning ensuing vocal actions and for a coherent representation of the environment in bats.*

Minor comments:

One of the difficulties in trying to digest this paper is the large number of analyses (e.g., different frequency bands at different depths in the FAF with different vocalization types, coherence at different frequencies and depths across brain areas). It is difficult to keep everything in perspective, to know what is important. The Discussion and the graphical abstract do a pretty good job summarizing the data.

Thank you!

p. 7, first para, last sentence: since the increase in neural activity BEFORE the vocalization is not significant, this statement should be removed.

You are right. We removed the sentence.

Section on SVM classifier is neat but think can be moved to supplementary information section for create space for additional analyses (see Major Comments). I also think the section on the relationship between spiking activity and the LFP is interesting, but see it as secondary to the main point of the paper; so this section too could be moved to the supplementary material (or streamlined substantially).

Thank you for this suggestion. We would prefer to keep the SVM classifier analysis within the main manuscript, especially since we added more information to this figure obtained based on the referee’s suggestions using post-vocalization responses and calls with contaminated post-vocalization time (Fig. 6b and c of this MS version).

Following your suggestions, we reduced the part of the text dealing with LFP-spike coupling. In addition, inter-areal spike-LFP phase coupling was removed completely (panels (g-l) of former Figure 8 and (g-l) of former Supp. Fig. 8; in the current MS version Fig. 9 and Supp. Fig 11, respectively).

Figure 5. Why have two different depictions of accuracy across CN and FAF? Why not depict the data for CN as a heat map and add as a row onto the heatmap for FAF? Would save space and reduce the amount of work that readers have to do

Thank you for this suggestion. We added the CN as heatmap above the FAF. This figure has also grown since we ran the SVM analysis on the post-vocalization dataset and on the prevocalization dataset with contaminated post-vocal activity (Fig 6b and c of this MS version).

Please explain why opening and closing the recording chamber stimulated vocal production.

Thanks for this question. We did not quantify this method thoroughly, but one possible explanation could be that opening and closing the recording chamber (for just a few seconds) changed the immediate surrounding of the bat. This could have increased the bat’s “curiosity”, but this is only a speculation.

Reviewer #3:

[identifies himself as Jagmeet S. Kanwal]

This study reports interesting and novel findings on the role of oscillations for production and perception of vocalizations in bats. These results are significant given our lack of understanding of the neural mechanisms underlying vocal control. The role of oscillations in neural activity during social communication is an active area of inquiry and this study extends recent findings. While the oscillations are interesting, their correlation with vocal production does not inform us of the causal mechanisms and circuit design. Nevertheless, the findings reported here could pave the way forward.

Technically, the study is well designed and executed on an important animal model in this field.

Data appear to be carefully analyzed in detail and all figures are generally well organized, but the writing of the paper can be improved, including proper use of terminology. One main point that stands out is that social vocalizations predominantly have low frequencies, whereas echolocation vocalizations are stereotypically high frequency. Therefore, the differences in neural oscillations may be directly related to the dominant frequency in vocalizations and only secondarily to the fact that one set is being used for navigation while the other is used for social communication. For a clear distinction into two functional types, it is essential to compare social vocalizations that have high frequencies within them with echolocation vocalizations. Until this is done, the differences in neural oscillations correlated with each type of output cannot be classified as relating to social versus navigational (echolocation). The authors may be able to parse out their social vocalization data to clarify this issue.

Alternatively, the distinction between neural oscillations can be related to predominant frequency within a vocalization, which may very well be the case.

Thank you very much for this insightful comment. The reviewer is right. There are communication calls that carry strong energy at high frequencies. Pooling together data from low and high frequency communication calls could hide possible mechanisms underlying the production of high frequency sounds not necessarily unique to echolocation.

Following the referee’s suggestion, we searched our data for high frequency communication sounds that could be directly compared to echolocation data. We were unable to find communication sounds containing only high frequencies, but we did find a subpopulation of communication calls containing high power at both low and high frequencies (LHF calls in this manuscript version, see example calls in Fig S2a-b).

Neural activity related to the production of LHF communication sounds was directly compared to echolocation as suggested by the referee and we obtained results similar to those reported when pooling all communication vocalizations together (Fig. 1f, g of this MS version). These results hint towards echolocation-specific processes in our data. Yet, we cannot discard that the production of purely high frequency communication sounds would involve neural activity similar to that reported here for echolocation. Readers are cautioned about the latter in the discussion, were we say (end of page 17):

*Based on spectro-temporal features we identified two types of communication vocalizations (containing either only low frequency or high and low frequency components, see Fig, 5 and Supplementary Figure 2). Even communication calls containing pronounced power at high frequency differed markedly from echolocation vocalizations. The latter indicates that differences observed in terms of LFP spectral structure and inter-areal coupling are not related solely to the absence/presence of high frequencies in the sounds uttered. Note, that we cannot discard that communication calls containing only high frequencies involve LFP patterns similar to those observed during echolocation. Communication calls carrying only high frequencies were not observed in our dataset, but C. perspicillata produces such calls in contexts such as mating [59].*

Since different types of intra- and into inter-areal oscillations are discussed, to help the reader, please summarize results in a table where the function and/or presence/location of each type of oscillation and its relationship to communicaton/echolocation or high/low frequency vocalization is listed.

Thank you for this suggestion. The table complements the graphical summary (now Fig. 10) and is listed as supplementary material (Supplementary Table S1).

Also, it is important to be clear that the reader does not think that neural oscillations play a direct role in vocal control; the neural oscillations are temporally correlated with different types of vocal output.

We agree on this. In this version of the discussion we emphasize that our paper shows only correlative evidence linking neural oscillations to vocal production. Specifically, we state this on p. 16 (end of 2^nd^ paragraph). We wrote:

*Note that this study presents only correlative evidence linking vocal production to neural oscillations in the fronto-striatal network. The latter does not imply a causal role of neural oscillations in vocal control.*

With regard to this, it will be useful to include a paragraph or section in the discussion about the origin of different types of oscillations in the brain in relation to this study (perhaps, expand on supplementary figure 9?). For example, high gamma oscillations are thought to result from the interaction of fast-spiking inhibitory with excitatory neurons within local neural circuits.

Also, oscillations observed in one region of the brain can originate elsewhere since they reflect synaptic activity. This can have a bearing on the proposed fronto-striatal connectivity.

Thanks for this insightful comment. We implemented the referee’s suggestion in the discussion (p. 16 (3^rd^ p.) to 17. We wrote:

*Neural oscillations are a generalized phenomenon in the nervous system, and they have been studied extensively in past years (for reviews see [29, 35, 62-64] ). The current consensus is that oscillations represent different excitability states in neural populations. Oscillatory activity differs across brain structures and it participates in processes such as inter-areal synchronization, local information binding, selective attention and memory formation, among others [27, 65, 66]. The neural mechanisms by which oscillations are generated in the brain are still under debate. At least gamma oscillations observed in the neocortex and hippocampus seem to originate from an interplay between excitatory and inhibitory activity in pyramidal cells and interneurons (PING networks, for review see [62, 67]). It has been postulated that oscillations in other frequency bands could also be linked to pyramidal-interneuron networks that oscillate with different time constants (see for example the PIN-theta networks proposed for the auditory cortex [64]). Low frequency oscillations (i.e. alpha and theta) also have been linked to pace-making pyramidal neurons, although it is not clear if the underlying mechanism for pace-making relates to pyramidal-interneuron networks as well [68, 69]. In humans, beta oscillations found in the striatum also seem to involve inhibitory interactions between neurons [70]. With our current data, we cannot assess the cellular mechanisms responsible for the oscillations observed in the bat striatum and frontal cortex during vocalization. Regardless of the cellular origin of fronto-striatal oscillations, our data shows that at least in bats, neural rhythms in these two structures correlate well with vocal output.*

Specific corrections:

Abstract:

First line: Change "… networks leading to vocalization production remain…" to "… networks underlying vocal control remain…"

Thank you. Done.

Change "... allows to assess the ... " to " ... allowed us to assess general... "

Thank you. Done.

Improve ".... the precise control of high gamma…" This is not good expression as the high gamma is not being controlled, but the vocal production is being controlled.

Thank you, that is right. We changed "... *the precise control of high gamma…*" to “*…a distinctive change in high-gamma…*”. (p. 2)

Change ".. Our data indicate.., " ("data" is plural).

Thank you. Done.

Change "... animals to access ... " to "... animals to selectively activate motor programs required for the production of either echolocation or social vocalizations. "

Thank you. Done.

Change to: "… animals are in a navigational mode (i.e., emitting echolocation pulses) or in a social communication mode (emitting social calls). Overall, this study presents…"

Thank you, we changed it to “*…animals are in a navigational mode (that is, emitting echolocation pulses) or in a social communication mode (emitting communication calls). Overall, this study indicates…*”

Introduction:

Change to "... motor output has not been clearly delineated." Also please report here the extensive work of Jurgens on neural pathways for vocal control in primates.

Thank you. We changed the sentence and added references to the work of Jürgens in the second paragraph of the Introduction (Simonyan K, Jürgens U. 2003 and Jürgens 1982).

Clarify " a largely discussed how…". Something is missing here.

Thank you. We removed the “*a*” and corrected it to “*…it remains largely discussed how (and if) oscillations in neural networks…*”.

2nd para: here and elsewhere, delete "production". "Vocalization" implies production.

Thank you. We removed “*production*” from “*vocalization production*” throughout the manuscript.

Clarify "associative circuit" - does not seem to be directly relevant here.

Thank you for this comment. We removed the term “*associative circuit*” from the manuscript as this term is not very relevant for understanding the paper.

To answer your question, there are five known fronto-striatal circuits, with the associative circuit being one of them. They connect different parts of the prefrontal cortex with the striatum (such as e. g. the dorsolateral PFC with the dorsolateral caudate (“associative circuit”) or the ventral striatum with the lateral orbitofrontal cortex (“limbic circuit”)). Despite the differences in anatomy. They also seem to hold (partially) different functions, e.g. the associative circuit seems to be predominantly involved in high-order cognitive functions, decision making, working memory and executive control, whereas e. g. the limbic circuit is critical for emotional processing and reward-based behavior.

"... latter could suggest…" – Delete "could"

Thank you. We decided to rewrite the second paragraph of the introduction and deleted the sentence in question.

P. 4: Here and elsewhere: Avoid use of the term "echolocation calls" and "non-echolocation calls"

Also, "Non-echolocation calls such as communication, distress and social calls". This is confusing. Better to have clear separation and consistently use "echolocation pulses or vocalizations". Non-echolocation vocalizations should instead clearly refer to "social calls". Distress calls also fall under the category of social calls. These are the only two well-recognized types of vocalizations. Non-echolocation calls is a functionally diffuse category.

Thank you for this important comment. We changed “echolocation calls” to “echolocation pulses” throughout the manuscript. The “non-echolocation calls” were changed to “communication calls” as suggested by Reviewer 1. We would prefer this term, but could also change it to “social calls” if needed. Note that other articles dealing with vocal production have also used the term communication calls to refer to non-echolocation sounds (see Frenzl & Schuller BMC Biology 2005; Fenzl and Schuller Behavioural brain research 2007).

P. 7: Please briefly indicate the importance of Cliff's delta metric and why it was used here to compare effect size.

Thank you for this comment. We added the following (p. 9 , end of 2^nd^ paragraph)

“*Briefly, the d-metric describes the effect size of group comparisons and ranges from -1 to 1 with identical groups rendering values around zero [60]. This measure was designed for non-parametric tests (in contrast to Cohen’s d) and it quantifies how often values in one distribution are larger than values in a second distribution.”*

Change to: "… before call production compared to before emission of echolocation pulses…"

Thank you. Done.

Last sentence on page 11 is not clear.

Not clear why the phase of LFPs in the FAF is compared to its relationship to striatal spiking. Please explain.

Thank you for pointing this out. The whole idea behind this analysis was to assess whether there is mutual influence of spiking-LFP behavior in both brain structures. As LFPs are thought to be able to mediate neuronal excitability, we wanted to test if the activity state in one brain structure correlates with the spiking activity of the other brain structure.

In any case, this inter-areal analysis was removed from the ms following suggestions made by reviewer 2 to reduce the part of the ms dealing with spiking.

Also, it is not clear why changes in spiking probability were not observed in response to vocalizations. Could this be because spiking activity was not recorded from relevant neurons given the sparse nature of their distribution within the FAF?

Yes, you are right. The fact that we did not observe an increase in spiking probability related to vocalization production/hearing could be due to their low density in the FAF. We mention this point in the figure legend (Fig. 8 in this ms version).

Discussion:

As used elsewhere, change "diseases" to "disorders".

Thank you. Done.

Last sentence on page 12: please state more clearly. Which motor action? Also, visual abstract in supplemental figure 9 can be improved in terms of labels and colors and clarity in general.

Thank you. We changed motor action/motor output to “selecting and producing different types of vocalizations” and “vocal motor outputs”.

In the summary figure, we changed the colours, labels, and highlighted important parts. This figure is now part of the main manuscript (Fig. 10) but it refers as well to the supplementary table suggested by the reviewer (Supplementary Table S1).

P. 14: please provide some reasoning for the first sentence in the second paragraph on this page.

Thank you. With this sentence, we were referring to the results of the coherence analysis, where higher coherence between the top layer of the FAF and CN was observed in the theta band.

We changed the sentence to (p. 18, 3^rd^ paragraph)

*“Our data suggests that low frequency rhythms in frontal areas (especially theta, see Fig. 7) relate to inter-areal communication between FAF superficial layers and the dorsal striatum during vocalization, as quantified by computing inter-areal LFP coherence (Fig. 7). This result falls in line with a putative involvement of low frequency oscillations in long range synchrony [27, 28]. The FAF constitutes a non-classical sensory area and its laminar structure (i.e. location of inputs and outputs, such as layer 4 and 5 in sensory cortices [75]) needs further anatomical exploration.*”

P. 16: change "component" to "outcome".

Thank you. Done.

P. 28, Figure 2 legend: "z-scored LFPs". Does this refer to the peak of each LFP?

No, it refers to z-scored LFP amplitude. Z-scoring was performed at each time point and channel. To preserve the relationship between LFPs across channels within each recording site, we z-scored considering all signals recorded simultaneously across FAF layers.

P. 31: "... for different channels…". Should this be "... for all recorded channels..."?

Thank you. Done.
