# Supplementary material for: Neural oscillations in the fronto-striatal network predict vocal output in bats
Source: PLoS Biol. 2020 Mar 19;18(3):e3000658. doi: 10.1371/journal.pbio.3000658 (PMC7081985; doi:10.1371/journal.pbio.3000658)
Supplement: Attachment — Submitted filename: response letter.docx [file pbio.3000658.s014.docx]

Response to editorial comments: responses are in black, comments in red

1.	Please note that your submission cannot contain any references to generic "supporting information" files and instead must refer to specific files using our naming convention (S1 Fig, S1

Table, S1 Text, S1 Data, etc.)

corrected

2.	Many thanks for providing the data in g-node. However, in order to make the Figures more standalone, please could you clearly cite the DOI in all of the relevant Figure legends (e.g. "Data underlying this Figure can be found at DOI: 10.12751/g-node.6a0d94"). I believe this applies to Figs 1-9, S2-S8, S10, S11.

Done. The DOI was added to the caption of figures 1-9, S2-S8, S10 and S11.
